# Editorial: DNA methylation in plants associated with abiotic stress, volume II

**DOI:** 10.3389/fpls.2023.1203806

**Published:** 2023-05-24

**Authors:** Markus Kuhlmann, Hua Jiang, Marco Catoni, Frank Johannes

**Affiliations:** ^1^ Department Molecular Genetics, RG Heterosis Leibniz Institute of Plant Genetics and Crop Plant Research (IPK), Seeland, Germany; ^2^ Independent Research Group Applied Chromosome Biology, Leibniz Institute of Plant Genetics and Crop Plant Research (IPK), Seeland, Germany; ^3^ School of Biosciences, University of Birmingham, Birmingham, United Kingdom; ^4^ Department Molecular Life Sciences, Research Group Population Epigenetics & Epigenomics Technical University of Munich, Munich, Germany

**Keywords:** plant epidemics, DNA methylation, abiotic stress, RdDM, plants

In plants, methylation at the 5´ position of cytosines is an evolutionarily conserved epigenetic modification of nuclear DNA. The pattern of methylation at specific regions occurs with mechanisms of maintenance, *de novo* methylation and demethylation processes, which could be differentially modulated in response to environmental stimuli. Hence, the investigation of relationships between abiotic stresses and changes in DNA methylation patters is important to understand the role of epigenetics in the response to environmental challenges. In symmetric cytosine contexts (CG and CHG, where H stands for A, T or C), the DNA methylation pattern can be actively maintained from one cell generation to the next after replication using hemi-methylated sites as template. In addition, plants are the only organisms that display substantial methylation of cytosines in an asymmetric sequence context (CHH context). This *de novo* methylation mechanism involves the presence of small regulatory RNAs as triggering molecules for an RNA-directed DNA methylation (RdDM). The relationship of these dynamic methylation processes have been reviewed in relation to abiotic stress and plant development in two review published in this Research Topic, respectively by (Liu and He, 2020) and (Kumar and Mohapatra, 2021). DNA methylation is often associated with heterochromatic chromatin structures. Together with histone modifications, DNA methylation has a role in gene regulation, chromatin structuring, and repression of repetitive elements (Bhadouriya et al., 2020). This modification provides a heritable mark that can be propagated through mitosis and meiosis. The relevance of these mechanism in response to stresses in plants was discussed in this Research Topic.

Environmental factors can influence these patterns in several ways: First by direct regulation of genes involved in the methylation and demethylation processes, second by modulating the abundance of small heterochronic-RNAs (24mers). Their occurrence at different times, tissue or induced by environmental conditions is defining the region of methylation. Third, regulating the abundance of the methyl-group donor SAM which can contribute to effective maintenance and *de novo* methylation. Demethylation can occur either passive due to loss of methylation after replication or active due to active removal of methylated nucleotides due to DNA repair processes. Therefore a strong influence on DNA methylation patterns was also described for DNA damaging abiotic conditions. As DNA damage repair is strongly connected to DNA-demethylation processes a main focus is given in this Research Topic on antioxidant regulation (Cui et al.), connection to reactive oxygen species (ROS) (Jing et al.) but also heavy metals and copper ions (Bednarek et al.). These factors can potentially induce DNA damage and thereby modifying DNA methylation patterns.

As already pointed out in the editorial of the first volume of this Research Topic (Kuhlmann et al., 2021) the origin of most small RNAs is from repetitive DNA elements and retrotransposons, therefore it is obvious that environmental changes might lead to transcriptional reactivation of these elements and also *de novo* generation of heterochromatic small RNAs. These heterochromatic small RNAs can subsequently lead to DNA methylation in cis and trans homologous regions. Taking this into account, diverse stress conditions can induce *de novo* methylation patterns with suppressive function to gene activation.

One of the published work focuses on the crop plant *Triticale* (Bednarek et al.), a hybrid of wheat (*Triticum aestivum*) and rye (*Secale cereale*) widely used for food production. Therefore, regeneration of plants via tissue culture to obtain plantlets identical to the donor plants are economical of high interest. As result the authors found that *de novo* methylation is affected by the concentration of cooper in the media. Therefore, for many environmental changes, differentially methylated genomic areas or sites are described. In some cases, these changes are affecting nearby genes and can cause changes in the phenotype.

Two additional studies investigate dicotyledonous species of ecologic and economic importance for their remarkable fast growth: Pokeweed (*Phytolacca americana)* (Jing et al.) and Bitter wine (*Mikania micrantha*) (Cui et al.). In both species results are presented addressing changes in DNA methylation in relation to the production of reactive oxygen species as part of the plants response to abiotic stress. As reactive oxygen species are effectors of DNA damage, they are inducing the DNA damage repair pathway and thereby the removal of DNA methylation. The here presented manuscripts highlight the aspect of demethylation in plants that was not extensively covered in the first volume.

As already discussed in the first volume of this Research Topic (Kuhlmann et al., 2021) the study of repetitive DNA elements and retrotransposons should be considered in epigenetics studies, because they are at the origin of most small RNAs produced in plants, and their activation is regulated by epigenetic mechanisms. The ONSEN/COPIA78 family in *Arabidopsis thaliana* is the best example of environmentally-control retrotransposon, being specifically activated in response to heat stress (Ito et al., 2011; Nozawa et al.). In a study published in this Research Topic, Nozawa et al. found that ONSEN response to heat was enhanced in a natural Arabidopsis mutant accession of the epigenetic factor CMT2, a CHH methyltransferase involved in epigenetic silencing.

Collectively, this collection highlights the relevance of epigenetic response to abiotic stresses in plants in relation both to develop new strategies to improve plants and to study mechanisms of plant adaptation and evolution. We believe that this selection of works from volume II well integrates the previous volume, and it will round off the role of epigenetic in plants and can be of inspiration for future works in the same field.

## Author contributions

All authors listed have made a substantial, direct, and intellectual contribution to the work and approved it for publication.
